# Reproducibility of estimated optimal peak output using a force-velocity test on a cycle ergometer

**DOI:** 10.1371/journal.pone.0193234

**Published:** 2018-02-23

**Authors:** Manuel J. Coelho-e-Silva, Ricardo Rebelo-Gonçalves, Diogo Martinho, Alexis Ahmed, Leonardo G. O. Luz, João P. Duarte, Vítor Severino, Rafael C. Baptista, João Valente-dos-Santos, Vasco Vaz, Rui S. Gonçalves, Antonio Tessitore, António J. Figueiredo

**Affiliations:** 1 Research Unity for Sport and Physical Activity (CIDAF, UID/DTP/04213/2016), University of Coimbra, Coimbra, Portugal; 2 Faculty of Sport Sciences and Physical Education, University of Coimbra, Coimbra, Portugal; 3 Portuguese Foundation for Science and Technology, Lisbon, Portugal; 4 LACAPS, Federal University of Alagoas, Arapiraca, Brazil; 5 Faculty of Physical Education and Sport, Lusófona University of Humanities and Technologies, Lisbon, Portugal; 6 Department of Physiotherapy, Coimbra Health School, Polytechnic Institute of Coimbra, Coimbra, Portugal; 7 Department of Movement, Human and Health Sciences, University of Rome Foro Italico, Rome, Italy; Nanyang Technological University, SINGAPORE

## Abstract

The current study aimed to examine the reproducibility of estimated peak power and estimated pedal velocity in a multi-trial 10-s all-out cycling test among adult athletes (n = 22; aged 23.50±4.73 years). Stature, sitting height and body mass were measured. Leg length was estimated as stature minus sitting height. Body volume was obtained from air displacement plethysmography and was subsequently used to calculate body density. Fat mass and fat-free mass were derived. The short-term power outputs were assessed from the force-velocity test (FVT), using a friction-braked ergometer on two separated occasions. Differences between repeated measurements were examined with paired t-test and effect sizes calculated. No significant differences were found between session 1 (898 W, 142 rpm) and session 2 (906 W, 142 rpm). Test-retest procedure showed acceptable reliability for estimated peak power output [technical error of measurement (TEM) = 31.9 W; % coefficient of variation (CV) = 3.5; intra-class correlation coefficient (ICC) = 0.986] and pedal velocity (TEM = 5.4 rpm, %CV = 3.8, ICC = 0.924). The current study demonstrated a reasonable reproducibility of estimated peak power and pedal velocity outputs in non-elite male athletes and supports that a familiarization session including a complete FVT protocol is not required.

## Introduction

The understanding of anaerobic performance is partially contaminated by the use of different terminology such as supramaximal intensity exercise that was supposed to describe exercise intensities greater than those found at maximal aerobic power. Energy pathways do not operate independently and it is the type of motor activity, duration and intensity of the exercise that dictate which metabolic pathways are the predominant providers of adenosine triphosphate (ATP). In this context, the term maximal intensity should be accepted to mean “all-out” effort, irrespective of which energy system supplies the exercise. In the present study maximal intensity exercise refers to an “all-out” activity that is sustained by an anaerobic ATP yield which exceeds that of oxidative metabolism [1]. The main components of interest are peak and mean power outputs. Peak power (PP) refers to the maximum rate at which energy is produced and mean power corresponds to the total work done during the performance test divided by the amount of time (usually 30-s).

The Wingate anaerobic test (WAnT) is probably the most popular short-term maximal laboratory protocol [2]. It involves pedalling a cycle ergometer against a standardized braking force (Fb). Power is the product of force and velocity and, in the case of the cycle ergometer, each combination of Fb and pedal revolution may produce a different power output. However, the WAnT protocol related the Fb to a constant percentage of body mass and a Fb of 0.74 N.kg^-1^. An alternative to WAnT is provided by a set of all-out sprints against a range of Fb [3]. The force-velocity test (FVT) focuses on optimized PP and supposedly is not affected by the methodological constraints of the WAnT. It consists of a series of maximal 3–5 sprints performed against a range of Fb. A parabolic Fb power relationship and a quasi-linear Fb velocity relationship enable the individual determination of optimal velocity and Fb for estimated optimal PP (OPP) for each participant [4].

The FVT focuses specifically on PP and consists on short maximal sprints in the cycle-ergometer against several Fb’s. This is substantially different from other ergometer-based concurrent tests. For example, the WAnT protocol was designed to derive PP but also considers mean power (average output over 30-s) and permits a fatigue index. However, the WAnT adopts standardized Fb’s derived as a percentage of body mass which is a limitation assuming that power is a combination obtained from force and velocity. Although being classified as a promising test to assess short-term maximal intensity, the literature reporting data quality of the FVT is rather limited. A recent study [5] investigated the reproducibility of performance parameters obtained from 10-s maximal cycling effort against different Fb’s (4% to 11% of body mass) in young adult athletes (n = 48, males, aged 18.9–29.9 years) and concluded that reproducibility of measured PP tended to be acceptable and that within individual error was not related to Fb. The FVT provides a promising model for the investigation of short-term “all-out” assessment, but information about the reproducibility of optimal velocity, optimal Fb (OFb) and OPP was not abundant in the literature. Therefore, the purpose of this study was to examine the reproducibility of estimated optimal outputs derived from a force-velocity test performed on a cycle-ergometer. It was hypothesized that maximal cycling power computed from the parabolic relationship emerged from the application of a set of Fb’s and respective observed power outputs does not require a familiarisation session.

## Materials and methods

### Research design and procedures

The present study required repeated measurements of a maximal cycling power test with one week apart. Participants visited the Laboratory of Biokinetics located in Coimbra University Stadium for two occasions and were instructed not to eat for at least three hours before testing and not to drink caffeine before testing. All assessments were performed at the same hours of the day (10:00–12:00 a.m.).

### Participants

The sample was composed of 22 adults from different sports. Sample size was similar to previous studies aimed to examine the reproducibility of measuring cycling PP [6]. All participants were recruited in the Coimbra University Stadium according to the following inclusion criteria: males, aged between 19 years and 35 year-old; more than 2 years of training experience in competitive sports; none had any history of severe time-loss injury in the past six months; none were taking any medication or supplements known to affect performance. The research proposal was approved by the local Ethics Committee (Ethics Committee/Faculty of Sport Sciences and Physical Education-University of Coimbra/00102014). Procedures followed standards for research in sports medicine and were performed according to the Declaration of Helsinki. Participants were informed about the nature of the study, objectives, protocols and risks related to data collection. They provided written informed consent approved by the ethics committee before the beginning of the study. They were informed that their participation was voluntary and that they could withdraw from the experiment at any time. Verbal consent was provided during the second test session to ensure voluntary participation.

### Anthropometry

Anthropometric measurements inherent to size descriptors were performed by a single trained researcher following the protocols described by Lohman and colleagues [7]. Stature and sitting height were measured to the nearest 0.1 cm using a portable stadiometer (Harpenden model 98.603, Holtain LTD, Crosswell, UK) and sitting height table (Harpenden, Holtain LTD, Crosswell, UK), respectively. A portable scale was used to measure body mass (Seca model 770, Hanover, MD, USA) to the nearest 0.1 kg. A subsample (n = 13) was measured twice for the above mentioned variables to determine technical error of measurement (TEM) as recommended for Human Biology [8]. It was expressed in the same units as the variables and also as % of the pooled mean (%CV, coefficient of variation): stature (TEM = 0.37 cm; %CV = 0.21); sitting height (TEM = 0.71 cm; %CV = 0.81%) and body mass (TEM = 0.56 kg; %CV = 0.81%).

### Air displacement plethysmography

Body volume was derived from air-displacement plethysmography (Bod Pod^®^ Body Composition System, model Bod Pod 2006, Life Measurement, Inc, Concord, CA, USA). Before each trial, the precision of the equipment was tested using a 2-point calibration method using a 50.255-L cylinder and following the instructions of the manufacturer. Participants were assessed using lycra underwear and a swim cap as recommended by the manufacturer. Each individual seated in the chamber while the raw body volume was consecutively measured until two consecutive values within 150 mL were obtained. If more than three raw body volumes were necessary, the additional measurements were obtained after recalibrating the instrument. Body density as calculated as body mass (kg) divided by body volume (L) and was subsequently converted to percent fat mass using the equation proposed by Siri [9].

### Force-velocity test

The cycle ergometer (Monark 824E Peak Bike, Monark AB, Vargerg, Sweden) was calibrated according to the guidelines issued by the manufacturer before each test session. Prior to the test, participants completed a 5-minute standardized warm-up which consisted of pedalling with minimal resistance (basket supported) interspersed with 3-s “all-out” sprints at the second, third and fourth minutes followed by stretches of the quadriceps and hamstring muscles. The FVT implies a set of 3–5 maximal bouts of 10-s against a range of Fb’s (0.050 to 0.100 kg·kg^–1^; initial Fb set at 0.075 kg·kg^–1^ with subsequent Fb randomly above and below this intensity). The protocol began with minimal resistance (basket supported) at 60 rev ∙ min^-1^ and after a verbal sign “3-2-1-go”, participants started to pedal as fast as possible while the resistance was abruptly applied and the computer simultaneously activated. Data was obtained at a sample rate of 50 Hz. During each 10-s sprint, verbal encouragement was given for all participants. Sprints were interspaced by five minutes (active recovery pedalling at 60 rev.min^-1^ with minimal resistance). For each individual participant, OPP, OFb and optimal velocity were estimated [10].

### Statistical analysis

Descriptive statistics [minimum, maximum, mean, standard error of the mean, 95% confidence interval (95% CI) of the mean and standard deviation] were computed for the total sample on chronological age, training experience, body size and performance output obtained from the FVT protocol. The assumption of normality was checked. Subsequently, means and standard deviations were calculated for each time moment and also mean difference between session 1 and session 2 (including 95% CI of the differences). Differences between repeated measurements were examined with paired *t-tests*. Cohen’s *d* effect sizes and thresholds were used to evaluate the magnitude of differences [11]. Reliability was determined using technical error of measurement (TEM), coefficients of variation (%CV). In addition, Intra-class correlation coefficients (ICC) and respective 95% CI were determined. The Bland-Altman procedures [12] was used to plot each individual point based on the relationship among intra-individual differences and mean of the two occasions. Correlation coefficients were interpreted as follows [13]: trivial (*r* < 0.1), small (0.1 < *r* < 0.3), moderate (0.3 < *r* < 0.5), large (0.5 < *r* < 0.7), very large (0.7 < *r* < 0.9), nearly perfect (*r* > 0.9) and perfect (*r* = 1). Statistical significance was set at *p* < 0.05. The Statistical Package for the Social Sciences was used for the above mentioned analyses (SPSS v.22.0, Chicago, IL, USA).

## Results

Table 1 summarized descriptive statistics for chronological age, training experience, anthropometry and parameters extracted from the FVT (separately for sessions 1 and 2). Mean values for stature and body mass were also included in Table 1. They plotted around mean values for the Portuguese population. Estimated fat mass was 16.7%. Estimated PP output and estimated velocity derived from optimal load were apparently stable over time (Bias: 8 Watt; +1 rpm) with trivial intra-individual mean differences as presented in Table 2.

**Table 1:** Descriptive statistic for the total sample (n = 22).

Variable	unit	Range		Mean			Standard deviation	Shapiro-Wilk	
		minimum	maximum	value	SEM	(95% CI)		value	*p*
Chronological age	years	18.72	35,28	23,50	1,00	(21.40; 25.60)	4.73	0.828	<0.01
Training experience	years	2.00	27.00	9.86	1.26	(7.2; 12.5)	5.93	0.913	0.06
Stature	cm	165.1	179.3	172.9	0.91	(171.0; 174.8)	4.3	0.939	0.19
Sitting height	cm	87.9	96.8	92.3	0.6	(91.1; 93.5)	2.7	0.936	0.17
Leg length	cm	75.6	85.0	80.6	0.5	(79.5; 81.8)	2.6	0.970	0.71
Body mass	kg	49.5	104.9	70.4	2.8	(64.7; 76.2)	13.0	0.957	0.44
Fat mass	%	3.6	34.6	16.7	1.9	(12.8; 20.6)	8.7	0.941	0.21
Fat mass	kg	2.1	36.6	12.1	1.7	(8.5; 15.7)	8.2	0.871	<0.01
Fat-free mass	kg	38.8	72.5	58.0	2.1	(53.7; 62.3)	9.6	0.887	0.02
Session 1									
Estimated PP-OFb	Watt	500	1272	898	41	(812; 983)	193	0.936	0.163
Estimated V-OFb	rpm	113	169	142	3	(136; 148)	14	0.977	0.858
Session 2									
Estimated PP-OFb	Watt	497	1268	906	42	(819; 992)	195	0.944	0.239
Estimated V-OFb	rpm	185	142	142	3	(135; 149)	15	0.939	0.189

Abbreviations: SEM, standard error of the mean; 95% CI, 95% confidence intervals; PP-OFb, peak power optimal braking force; V-OFb, velocity optimal braking force.

**Table 2:** Means and standard deviations for session 1 and session 2, mean differences between time-moments including 95% confidence intervals, paired t-test and effect size (n = 22).

Variable	unit	Session 1	Session 2	Mean difference		*t*	df	*p*	Magnitude effect	
				value	(95% CI)				d	(qualitative)
Estimated PP-OFb	Watt	898 ± 193	906 ± 195	-8	(-28; 12)	-0.831	21	0.416	0.18	(trivial)
Estimated V-OFb	rpm	142 ± 14	142 ± 15	1	(-3; 4)	0.305	21	0.764	0.07	(trivial)

Abbreviations: 95% CI, 95% confidence intervals; PP-OFb, peak power optimal breaking force; V-OFb, velocity optimal breaking force; t, paired t-test; df, degrees of freedom; d, Cohen’s *d*.

**Fig 1:**
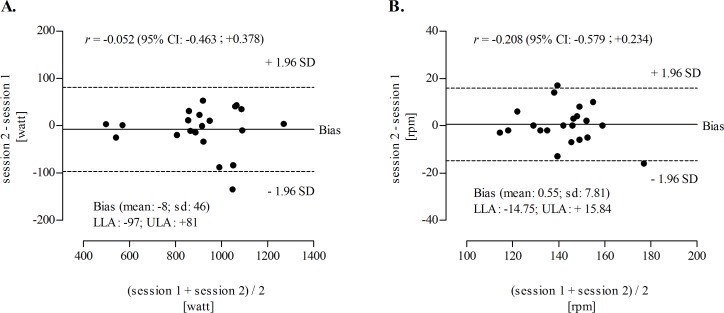
**Agreement of repeated measures for peak power optimal braking force (PP-OFb; panel A) and velocity optimal braking force (V-OFb; panel B).** The relation between residuals (absolute mean differences between session 2 and session 1) and the corresponding mean (heteroscedasticity diagnostic) are also presented [95% confidence intervals (95% CI)]. The dashed lines represent 95% limits of agreement (±1.96 SD); lower limits of agreement (LLA) and upper limits of agreement (ULA).

Reproducibility statistics for estimated PP output derived from optimal load (PP-OL) and estimated pedal velocity (V-OL) were presented in Table 3. Technical error of measurement was 31.9 Watt and 5.4 rpm, respectively for PP-OL and V-OL with %CV less than 5% for both variables. Meantime, intra-class correlation coefficient was 0.986 (95%CI: 0.966; 0.994) for PP-OL and 0.924 (95%CI: 0.818; 0.969). Finally Fig 1 illustrated the discrepancies of repeated measurement (Y axes: session 2 minus session 1) for PP-OL (panel A) and V-OL (panel B). The panels did not suggest heterocedasticity among axes (with X-axes being the mean of the repeated measurement) neither by visual inspection of the graphic nor based on statistics. Correlation coefficient among X and Y were -0.052 (95%CI: -0.463; +0.378) for PP-OFb and -0.208 (95%CI: -0.579; +0.234) for V-OFb.

**Table 3:** Correlation between trial sessions, technical error of measurement (TEM), coefficient of reliability (*R*), coefficient of variation (%CV) and intra-class correlation coefficient (ICC) (n = 22).

Variable	unit	Reliability		ICC	
		TEM	%CV	value	(95% CI)
Estimated PP-OFb	Watt	31.9	3.5	0.986	(0.966; 0.994)
Estimated V-OFb	rpm	5.4	3.8	0.924	(0.818; 0.969)

Abbreviations: 95% CI, 95% confidence intervals; PP-OFb, peak power optimal breaking force; V-OFb, velocity optimal breaking force; TEM, technical error of measurement; %CV, coefficient of variation; ICC, intraclass correlation coefficient.

## Discussion

Most studies of cycling power in children and in adults have used the popular 30-s WAnT. Few authors have reported the reproducibility of PP output measurements [5, 14]. This study hypothesized that maximal cycling power computed from the OL derived from the parabolic relationship between Fb and power output does not require a familiarisation session. Results confirmed that estimated PP-OL obtained from the FVT protocol seemed to be reasonable reproducible in adult non-elite athletes. A previous study [5] examined reproducibility of 10-s PP measured in cycling effort against different Fb’s in male young adult athletes (n = 48) and reported a TEM of 59 Watt (%CV = 5.52%; ICC = 0.951, 95% CI: 0.912; 0.972). Early studies using the WAnT recommended the determination of PP output over a 5-s period and assumed that this would reflect alactic anaerobic performance [15]. Subsequent research in adults evidenced a substantial increment in the concentration of muscle lactate during the first seconds of the maximal effort [16]. In addition, mean values of PP were relatively stable when obtained from sampling rates of 50 Hz or while corresponding to a sampling rate of 1 Hz [5]. However, the FVT has assumptions such as the quasi-linear Fb velocity and parabolic Fb for the determination of PP-OL and V-OL, and potential error of measurement associated to Fb’s would theoretically affect the interpolation of PP-OL and V-OL [14]. The current study concluded that those parameters were reasonable stable with trivial intra-individual mean differences between time-moments, no evidence of heterocedasticity among intra-individual values and mean of the two measurements and residual bias (8 Watt for PP-OL and 1 rpm for V-OL).

A reproducibility study comprising 27 pre-pubertal children aged 9.8 years who completed 3–5 sprints using Fb’s between 1.5% and 7.5% of body mass. The mentioned study did not observe learning effect between tests 1 and 2, but when Fb was less than 5% of body weight, the reproducibility of the test was lower [6]. The authors also tested adults and concluded that the highest Fb (10% of body mass) also negatively affected the reproducibility coefficients and, for adults, to obtain reproducible measures of cycling PP, a familiarization session including a complete test protocol was recommended [6]. In the current study, intra-individual mean differences were negligible and it seemed that an introductory session for familiarization is not required for adults, at least among adult athletes. Consequently, generalization of conclusions should be done with caution and the recommendations of the present study would be valid for non-elite athletes within the specific characteristics for sex, chronological age, training experience, body size and composition. The principal disadvantage of the FVT is probably the amount of time needed for completion of the protocol in comparisons to concurrent anaerobic tests (WAnT, repeated running sprint ability, 140-m shuttle run in the basketball court) [5, 17]. Meantime, it would be possible to advocate the use of the FVT an auxiliary tool to obtain the optimal load for a 30-s sprint as in the WAnT [14]. The current study demonstrated a reasonable reproducibility of PP-OL and V-OL and supports the adoption of the FVT in its own and not just a prerequisite for another test.

Predictors of short-term maximal testing highlighted the interdependent of the outputs with body size descriptors [10, 17]. About 52% of the inter-individual variance in peak output obtained by the WAnT was explained by non-invasive indicators of biological maturation that is strongly related to fat-free mass and also by estimated leg length and body mass among adolescent basketball players aged 14–16 years [17]. By inference, setting a Fb in relation to body mass when performance is strongly associated with muscle mass is a recognized limitation for WAnT and FVT [3, 14, 17, 18]. Based on the data of the current study, it was possible to search for potential correlates of mean performance (sum of values obtained from the sessions divided by two). Body mass and fat-free mass were largely and significantly correlated with mean performance derived from repeated tests (body mass: r = +0.728, 95%CI: +0.507; +0.939; fat-free mass: r = +0.882, 95%CI: +0.771; +0.959). Meantime, intra-individual error (session 2 minus session 1) was not significantly correlated to fat-free mass (r = +0.004, 95%CI: -0.322; +0.495) neither to body mass although the magnitude of the coefficient was small (r = -0.335, 95%CI: -0.627; +0.382). Developmental changes in OPP in boys and girls aged 12–14 years were largely explained by increases in thigh volume [14]. The FVT was performed in a seated position and consequently it would be hypothesized that performance and error would be more dependent from regional size descriptors such has leg length and thigh volume [1, 10, 14]. In the current data correlation of mean performance (sessions 1 and 2) was moderate with the appendicular size descriptor (leg length: r = +0.353, 95%CI: -0.074; +0.683). The magnitude of the correlation of error (session 2 –session 1) and leg length was -0.132 (95% CI: -0.502; +0.323). In other words, fat-free mass from the whole body was the best single predictor of inter-individual and was the unrelated to error.

The present study has several limitations that should be mentioned. The sample size could be larger and not of convenience. Moreover, only three sprints were considered and the literature often mentions 3–5 trials to compute the parabolic function between Fb and power [1, 5, 6, 14]. Consideration of a third evaluation session with 1-week apart could be informative to truly understand the effect of familiarization between sessions 1 and 2 or possibly between sessions 2 and 3.

Meantime, the results of the present study are possible to be integrated in the available literature [1–5, 10, 14–18]. During daily physical activity, people are more often engaged in short-burst than long-burst exercise and in many sports, especially team sports, assessment of short-term muscle power is important [1, 10, 17]. The 30-s friction-braked cycle ergometer protocol, termed WAnT, is probably the most used cycle ergometer protocol [1–3, 15–18]. The original research adopted the same Fb for all participants, but the subsequent versions of the test have related the Fb to body mass [1–4]. The load originally proposed was 7.5% of body mass. Subsequently, researchers discussed the load commonly used for this test and argued that it was too low or too high and, as a result, different load applications were recommended to obtain the greatest power output [5, 6, 14]. The FVT protocol assumes a quasi-linear relation between Fb and angular velocity, and a parabolic function between Fb and power [14]. A reliable test allows confidence in the monitoring of changes that occur during a season. It was previously suggested that if a cycling familiarization session was not performed, the data quality of the maximal cycling power was affected in adults but not in children [6]. However, intra-individual variation was examined from repeated tests within one week and results adult athletes aged 18–29 years suggested that reproducibility of PP obtained from single 10-s trials tended to be acceptable and intra-individual error appeared unrelated to Fb [5]. Consistently, the current study examined maximal cycling power computed from the parabolic relationship emerged from the application of three Fb and respective observed power outputs and concluded that the test does not require a familiarisation session. This information has practical implications in the management of research projects, particularly for longitudinal assessments.

Future research may examine the agreement between estimated PP computed from the FTV and measured using the WAnT protocol with estimated optimal load derived from the FVT. Peak power in cycling test is generally obtained over 1-s to 5-s epochs and with the relative ease and popularity of computer driven data collection systems, estimates of PP over several time periods are possible [1, 5, 6, 14, 17]. It would be relevant to replicate the findings of the present study using different sampling rates.

## Conclusions

The current study hypothesized that maximal cycling power computed from the parabolic relationship emerged from the application of a set of Fb’s and respective observed power outputs does not require a familiarisation session. The results evidenced that estimated PP-OL and V-OL outputs obtained from the FVT seemed to be reasonable reproducible in adult non-elite athletes.

## Supporting information

S1 File Full dataset. (XLSX)
